# From Localization to Coordination: Distributed Causality and the Emergence of Biological Function in the Brain and Plant Systems

**DOI:** 10.3390/biology15120936

**Published:** 2026-06-15

**Authors:** Umberto Castiello

**Affiliations:** Department of General Psychology, University of Padova, Via Venezia 8, 35131 Padova, Italy; umberto.castiello@unipd.it

**Keywords:** brain networks, connectome, effective connectivity, dynamic causal modeling, plant signaling, cross-talk, systems biology, emergence, distributed causality

## Abstract

The classical localizationist framework in biology and neuroscience has provided a powerful approach for linking structure to function. However, increasing evidence indicates that biological functions emerge from distributed interactions across complex systems. While network and systems-based approaches have advanced this transition, they often remain focused on connectivity patterns or statistical dependencies. In this review, I argue that a further conceptual step is required: a coordination-based framework in which biological function emerges from the context-dependent selective stabilization of interactions among distributed components that become causally relevant for specific outcomes. I develop this perspective comparing brain network organization and plant signaling, two systems that exhibit adaptive behavior without relying on centralized control. Across both domains, function depends on the dynamic coordination of heterogeneous processes operating across multiple spatial and temporal scales. This framework acknowledges structural specialization but argues that specialized components become effective through coordinated interaction regimes. I further discuss how this perspective extends current systems biology approaches by prioritizing temporally structured interaction patterns as the primary explanatory target. Finally, I outline empirically testable predictions suggesting that biological function is better captured by time-resolved coordination dynamics, hub-mediated integration, and metastable interaction regimes than by localized activity or static connectivity.

## Simple Summary

Scientists have long believed that different parts of the brain or body are responsible for specific functions. However, growing research shows that living systems work more like teams than isolated parts. This article explains that both the human brain and plants depend on many signals and processes working together in a coordinated way. For example, the brain creates thoughts and behavior through communication between many regions, while plants can react to stress, injury, or changes in the environment by sending signals throughout the whole organism, even though they do not have a brain or nervous system. The study suggests that what matters most is not where a function is located, but how different parts interact and cooperate over time. By comparing brains and plants, the article shows that complex behavior can emerge without a single control center. Understanding living systems in this way could help scientists better study health, behavior, and adaptation in both humans and plants, and may lead to new approaches in biology and neuroscience research.

## 1. Introduction

The localization of function has historically provided a powerful framework for linking brain structure to behavior. From early lesion studies to modern neuroimaging, identifying discrete regions and networks associated with specific cognitive processes has shaped neuroscientific inquiry. However, increasing evidence suggests that this framework alone cannot account for the flexibility, redundancy, and context-dependence of cognition [1].

Network neuroscience has demonstrated that cognitive processes depend on interactions among distributed brain regions organized into large-scale functional networks, including the default mode, salience, and frontoparietal systems [2,3]. These networks dynamically reconfigure across time and task demands, indicating that brain function cannot be reduced to static anatomical localization [4].

**Figure 1:**
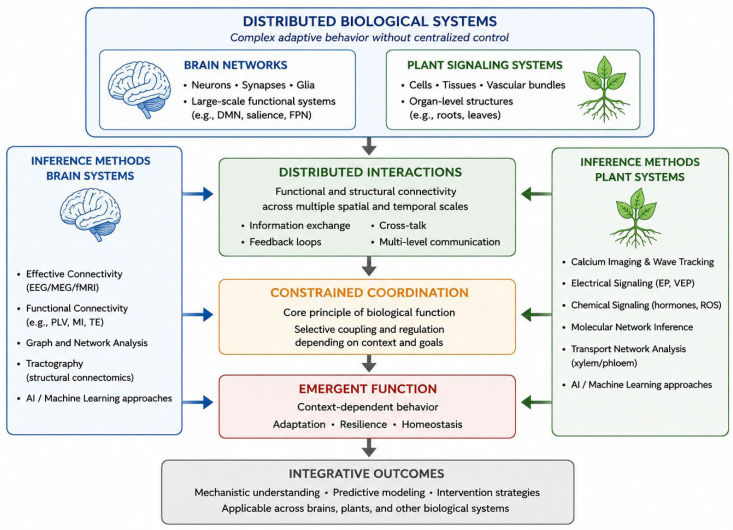
Conceptual framework of coordination as a general principle of biological organization. Biological systems, including brain networks and plant signaling systems, are composed of distributed components that interact across multiple spatial and temporal scales. These interactions give rise to constrained coordination regimes, defined by context-dependent selective coupling and regulation, which constitute a core principle of biological function. Such coordination enables emergent, adaptive behavior without requiring centralized control. The framework integrates complementary inference methods developed for brain systems (**left**) and plant systems (**right**) to reconstruct functional and structural interactions. Arrows indicate bidirectional conceptual links between inference approaches and the characterization of distributed interactions in each domain. EEG, electroencephalography; fMRI, functional magnetic resonance imaging; MEG, magnetoencephalography; PLV, phase-locking value; MI, mutual information; TE, transfer entropy; EP, extracellular potential; VEP, variation in electrical potential; ROS, reactive oxygen species; DMN, default mode network; FPN, frontoparietal network.

Yet, the shift from localization to distribution remains incomplete if interpreted purely in structural terms. A distributed system does not necessarily explain how coherent function emerges. The central question is therefore not whether the brain function is localized or distributed, but how interaction regimes become coordinated into functionally meaningful patterns. Here I propose that coordination, rather than localization or distribution alone, should be regarded as the primary explanatory principle of biological function. Coordination refers to the context-dependent selective organization of interactions into transient regimes that constrain system-level outcomes [5,6,7]. Unlike approaches that primarily characterize connectivity patterns or network states, the present framework focuses on how interactions become functionally effective under specific conditions. This conceptual shift is summarized in Figure 1.

## 2. Limits of Localization and the Need for Coordination

Operationally, coordination can be defined as the selective stabilization of interaction patterns among system components that are causally relevant for producing specific functional outcomes in a given context. Crucially, coordination is not equivalent to temporal variability in interactions, nor to the mere presence of dynamic functional connectivity. Rather, it refers to those subsets of interactions that are both context-sensitive and functionally effective, in the sense that their organization contributes to the generation, maintenance, or modulation of system-level behavior.

A concrete example can be found in magnetoencephalography (MEG) studies of large-scale neural coordination. During cognitive tasks, distributed cortical regions transiently synchronize within specific frequency bands over limited temporal windows [5,8]. Such coordination can be quantified using measures including phase-locking value (PLV), mutual information, transfer entropy, or time-resolved functional connectivity analyses [4,9,10]. In this context, coordination does not refer to generic fluctuations in connectivity, but to selectively stabilized interaction patterns whose emergence predicts behavioral performance or cognitive state transitions [7,8]. Similar approaches have also been used in resting-state analyses to identify meta-stable coordination regimes that dynamically reorganize across time [5,6,11].

This distinction implies that not all time-varying interactions constitute coordination. Dynamic functional connectivity captures fluctuations in statistical dependencies between signals, but does not by itself establish which interactions are functionally consequential. By contrast, coordination requires the identification of interaction patterns whose selective stabilization predicts or constrains functional outcomes, and whose perturbation leads to systematic changes in system behavior. This perspective is compatible with intervention-based accounts of causation, according to which causal relevance is established by the extent to which perturbations selectively modify system behavior under controlled conditions [12].

From an empirical perspective, coordination can therefore be operationalized as the subset of time-resolved interactions that (i) exhibit context-dependent selection, (ii) contribute to cross-context prediction of functional outcomes, and (iii) show causal relevance under perturbation. This definition differentiates coordination from descriptive measures of network dynamics and provides a basis for testing it against alternative accounts based on static connectivity or undifferentiated temporal variability.

While localization captures important aspects of brain organization, including regional specialization and structural constraints, it fails to explain several key phenomena. Among these are degeneracy, the ability of structurally different elements to perform similar functions [13], and context-dependent functional recruitment, whereby the same region participates in different processes depending on network configuration [14].

These observations suggest that function cannot be fully identified with anatomical location. At the same time, abandoning localization entirely would ignore the biological constraints imposed by structure. The challenge is therefore to move beyond a purely spatial ontology of function without losing mechanistic specificity.

A coordination-based framework addresses this issue by defining function as the outcome of structured interactions among components, rather than as a property of individual regions. In this view, brain regions contribute to function not by virtue of their isolated activity, but through their participation in dynamically organized interaction patterns. The novelty of the coordination framework does not lie in proposing additional forms of network dynamics, but in redefining the primary explanatory unit. Connectivity, metastability, and dynamic coupling become explanatory only insofar as they contribute to functionally effective coordination regimes. In this sense, metastability describes a dynamical property of biological systems, whereas coordination refers to the selective organization of interactions that becomes causally relevant for specific functional outcomes.

## 3. Distributed Causality in Brain Networks

The concept of the connectome has reframed the brain as a networked system in which connectivity plays a central role [15]. However, not all connections are equally relevant at all times. Brain function depends on the selective engagement and modulation of specific pathways.

Effective connectivity approaches, particularly Dynamic Causal Modeling (DCM), provide a computational framework relying on Bayesian inference to identify and quantify causal interactions between brain regions, that is, how activity in one area influences activity in another over time [9,16]. These models reveal that causal influence is distributed across networks and dynamically reconfigured according to context and/or task demands.

Similarly, studies of dynamic functional connectivity show that large-scale networks fluctuate over time, even at rest, forming transient states that support different cognitive processes [4,5]. Hub regions, such as those in the frontoparietal cortex, play a critical role in integrating information across networks [17].

For example, large-scale networks such as the default mode network and salience network [3,17] dynamically reconfigure across cognitive states, illustrating how distributed regions are coordinated into functionally coherent systems.

Importantly, brain structural connectivity reconstructed through MRI tractography (DTI) provides only a partial and sometimes ambiguous representation of underlying pathways, with known limitations in resolving crossing fibers and accurately identifying long-range connections [18,19]. It should be noted, however, that the problem of crossing and kissing fibers is, at least in principle, addressed by more advanced diffusion imaging techniques such as Diffusion Spectrum Imaging (DSI). Unlike conventional DTI, which assumes a single dominant fiber orientation per voxel, DSI reconstructs the full diffusion orientation distribution function, enabling the resolution of multiple fiber orientations within the same voxel. This makes it theoretically capable of capturing complex configurations such as crossing, kissing, and branching fibers, although practical limitations remain in terms of acquisition time, signal-to-noise ratio, and biological validation [20,21,22]. This underscores that connectivity itself is not directly observed but inferred. In this context, a relevant contrast emerges when comparing brain connectivity with plant signaling systems.

This suggests that the key difference between brain and plant systems does not lie in the presence versus absence of inferential reconstruction, but in the degree to which interaction dynamics are directly observable. While brain connectivity is largely inferred from statistical dependencies in neuroimaging data, plant systems often provide more immediate access to propagating signals. Nevertheless, in both domains, functional organization emerges from patterns of interaction that must be interpreted rather than directly observed. This reinforces the view that biological function is intrinsically relational and must be reconstructed through inferential methods. The greater observability of propagating signals in plants may nevertheless provide empirical advantages for tracking interaction dynamics.

## 4. Plant Signaling and Systemic Cross-Talk

Plant systems provide a compelling example of complex coordination without centralized control. By “plant systems” I refer to the hierarchical and functionally integrated organization of the plant body, in which cells form tissues, tissues form organs, and organs are coordinated within broader organ systems, such as the root and shoot systems. These systems are connected through vascular and signaling pathways and operate without a centralized control structure, relying instead on distributed physiological coordination. Unlike animals, plants lack a nervous system, yet they exhibit highly integrated responses to environmental stimuli.

Systemic signaling in plants involves the propagation of electrical signals, calcium waves, reactive oxygen species (ROS), and hormonal pathways across the organism [23]. For instance, local stress can trigger rapid systemic responses mediated by glutamate receptor-like channels and calcium signaling, leading to coordinated defense activation [24].

Hormonal pathways, including auxin, jasmonate, ethylene, and salicylic acid, interact extensively, forming a network of cross-talk in which signaling pathways modulate one another’s activity [25]. These interactions enable plants to integrate multiple environmental cues and generate context-dependent responses.

From a network perspective, plant signaling systems can also be described in terms of nodes and interaction pathways. Functional nodes may include individual cells, vascular tissues, signaling hubs, or organ-level structures such as roots and leaves [23,25,26]. Interaction pathways correspond to calcium-wave propagation, reactive oxygen species diffusion, plasmodesmatal connectivity, electrical signaling, and long-distance transport through xylem and phloem tissues [23,24,27,28,29,30]. Although these pathways differ mechanistically from synaptic connectivity in neural systems, they similarly enable distributed communication and systemic coordination across multiple spatial scales [25,31,32].

For instance, systemic calcium waves can rapidly propagate across plant tissues following local stimulation, coordinating organism-wide responses through integrated signaling pathways. A representative example is vascular tissue, which includes xylem and phloem. Xylem mediates the transport of water and mineral nutrients from the roots to aerial organs, whereas phloem distributes sugars, metabolites, and signaling molecules throughout the plant. This tissue system not only enables long-distance transport, but also contributes to the coordination of distant organs by integrating physiological and signaling processes across the organism. A concrete example of such coordination can be observed in plant wound responses. Local tissue damage triggers rapid calcium waves that propagate systemically [23,24], followed by the activation of ROS signaling, which amplifies and relays the signal across tissues [23,27]. These fast signals are subsequently integrated with slower hormonal pathways, such as jasmonate signaling, leading to coordinated systemic defense responses [25,31]. Crucially, the functional outcome does not depend on any single pathway in isolation, but on the temporally structured interplay among these interacting processes.

Plant signaling should not be understood as the mere coexistence of multiple communication pathways [25,31], but as a temporally structured and functionally differentiated system. Electrical and calcium signals can mediate rapid long-distance propagation, reactive oxygen species can amplify and relay systemic responses [23,24,27], and hormonal pathways can modulate and stabilize these responses over longer timescales. In this sense, plant signaling is not simply distributed, but organized through the selective coordination of heterogeneous processes whose functional significance depends on physiological context and prior system state [26].

This perspective suggests that plant responses emerge from transient coordination regimes rather than from the activation of isolated signaling pathways [33].

Crucially, plant signaling is not organized around a central controller. Instead, coordination emerges from the integration of distributed signaling processes, demonstrating that complex adaptive behavior can arise from distributed interaction networks.

**Figure 2:**
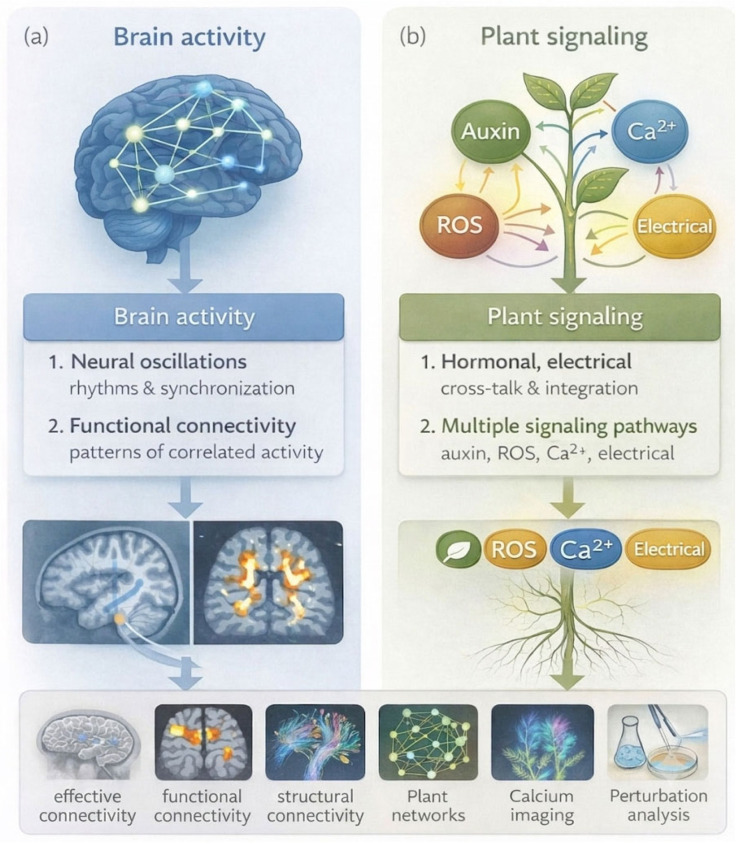
Comparative organization of distributed signaling in brain and plant systems. Brain activity (**a**) is characterized by distributed neural dynamics, including oscillatory activity and functional connectivity patterns, investigated using approaches such as dynamic causal modeling, tractography, and network analysis. Plant signaling (**b**) relies on cross-talk among multiple interacting pathways, including hormonal, electrical, calcium, and reactive oxygen species signaling. These processes are studied using network-based approaches, calcium imaging, and perturbation experiments. Despite differences in biological substrate, both systems exhibit distributed organization and require inferential methods to reconstruct their underlying relational architecture.

Notably, this organizational logic parallels observations in brain functional networks, where coherent outcomes emerge from the transient coordination of distributed processes rather than from the activity of isolated components [3,5,6]. In both domains, adaptive behavior arises through the self-organized integration of heterogeneous interactions across multiple spatial and temporal scales [34]. A comparison between these forms of distributed organization is illustrated in Figure 2.

### Limits of the Brain–Plant Comparison

The comparison between brain networks and plant signaling systems should not be interpreted as implying mechanistic equivalence between the two domains. Important differences remain in biological substrate, signaling speed, energy constraints, developmental organization, and evolutionary history [23,24,28,29,30,32]. Neural systems rely primarily on electrochemical signaling mediated by synaptic architectures and neurotransmitters [1,9,16], whereas plant systems coordinate responses through integrated electrical, calcium, reactive oxygen species, hydraulic, and hormonal signaling pathways [23,24,25,26,27,28,29,30,31]. Consequently, the comparison developed here is intended as a heuristic and organizational analogy rather than a direct functional mapping. The goal is to highlight a shared principle of distributed coordination in which coherent system-level outcomes emerge from transient and context-dependent interaction regimes without requiring a single centralized controller [6,11,34]. Despite these differences, both systems illustrate how coherent functional outcomes can emerge from context-sensitive interactions distributed across multiple spatial and temporal scales. The comparison is therefore intended to highlight a common organizational principle rather than a mechanistic correspondence. Accordingly, the comparison should be understood as organizational rather than cognitive, highlighting a common principle of distributed coordination without implying equivalent information-processing capacities.

## 5. Coordination as a General Principle of Biological Organization

While systems biology, network science, and network neuroscience have emphasized connectivity architectures, interaction patterns, and dynamic states, the coordination framework focuses on how specific interactions become selectively stabilized and causally relevant within a given context.

The comparison between brain networks and plant signaling systems suggests that distribution alone is not the defining feature of biological complexity. Rather, it is the organization of interactions into coordinated patterns that generates function.

In both systems, effective and contextually appropriate function emerges when interactions become selectively structured, forming transient regimes that integrate multiple components into coherent activity. These regimes are constrained by both structural connectivity and dynamic modulation of function. The selection of interaction patterns is not driven by a single agent, but emerges from the interplay between internal constraints and external conditions. Internal factors include structural connectivity, tissue organization, metabolic gradients, and intrinsic dynamics, while external factors include environmental inputs such as light, temperature, mechanical forces, and resource availability. Structuring is therefore not imposed top-down, but arises from reciprocal interactions among components across scales. Modulation is guided by feedback mechanisms, whereby ongoing system states influence the probability of specific interactions being stabilized, amplified, or suppressed.

This perspective aligns with systems biology approaches, which emphasize emergent properties arising from network interactions [35]. It also provides a framework for understanding how biological systems achieve robustness and flexibility simultaneously. A comparative overview of the main explanatory frameworks discussed in the present review is summarized in Table 1.

### Empirical Evidence for Coordination-Based Function

Recent empirical research increasingly supports the view that biological function emerges from the dynamic coordination of distributed interactions rather than from isolated components alone. Evidence from neuroscience and plant biology suggests that adaptive behavior depends on temporally structured patterns of interaction that continuously reorganize across changing internal and external conditions. These studies were not originally designed to test the coordination framework proposed here. Rather, they provide convergent evidence for its central prediction that functional outcomes depend on temporally structured interaction patterns rather than on isolated components alone.

In neuroscience, a growing body of work has demonstrated that cognitive processes depend on dynamic communication among distributed brain regions. Recent reviews have emphasized that understanding brain function requires characterizing how information is exchanged across large-scale networks rather than focusing exclusively on localized activity or static connectivity patterns [36,37]. These studies argue that brain organization is fundamentally relational and that communication dynamics play a central role in shaping functional outcomes.

Time-resolved analyses further support this perspective. For example, Vidaurre et al. [10] showed that spontaneous brain activity is organized into recurrent network states that evolve hierarchically across multiple temporal scales. Similarly, Shine et al. [8] demonstrated that cognitive performance is associated with transient transitions between integrated and segregated network configurations, indicating that successful behavior depends on flexible coordination among distributed neural systems. More recent edge-centric and time-resolved approaches have revealed that functional interactions continuously reorganize across time and behavioral contexts, providing further evidence that brain function depends on dynamic patterns of interaction rather than fixed architectures [38,39].

The importance of coordination is also supported by studies examining information integration across large-scale brain networks. Luppi et al. [40] identified a distributed “synergistic core” associated with higher-order cognition, suggesting that cognitive function emerges from integrated interactions among multiple regions rather than from localized processing alone. Consistent with this view, contemporary models of dynamic network connectivity emphasize that adaptive behavior depends on the flexible reconfiguration of interactions across distributed neural systems [41].

Comparable observations emerge from plant biology. Although plants lack centralized nervous systems, they exhibit highly coordinated systemic responses to environmental perturbations. Studies of long-distance signaling have demonstrated that local stimulation can trigger organism-wide responses mediated by electrical activity, calcium waves, reactive oxygen species (ROS), and hormonal signaling pathways [24,28]. These signaling mechanisms do not operate independently but interact across multiple spatial and temporal scales to generate coherent physiological responses.

Recent work has further highlighted the importance of integrated signaling networks in plant adaptation. Reviews of systemic communication emphasize that calcium, ROS, hydraulic, electrical, and hormonal pathways form highly interconnected signaling architectures whose functional significance depends on context and physiological state [42]. Such findings suggest that plant responses emerge from coordinated interaction regimes rather than from the activity of individual signaling pathways considered in isolation.

Taken together, evidence from both neuroscience and plant biology supports the view that biological function is best understood as an emergent property of transient and context-dependent interaction patterns. Although the underlying biological mechanisms differ substantially between neural and plant systems, both domains illustrate how adaptive behavior arises through the selective coordination of distributed processes. These observations provide empirical support for treating coordination as a central explanatory principle of biological organization (Table 2).

## 6. Methodological Convergence and Epistemological Implications

A crucial implication of the preceding comparison is that biological function cannot be directly observed, but must be reconstructed from indirect measurements. While the degree of observability of interaction dynamics may vary across systems, as in the contrast between brain connectivity and plant signaling, both domains ultimately rely on inferential frameworks to characterize functional organization.

In neuroscience, effective connectivity infers causal interactions from neuroimaging data, while tractography reconstructs structural pathways from intensity signals arising from water molecules moving in neural tissues. These approaches rely on models and are subject to uncertainties and biases [9,19]. This has direct implications for experimental design, as different measurement techniques may lead to different inferred organizations of the same underlying system. The limitations of diffusion MRI tractography discussed above illustrate how inferred connectivity depends on methodological assumptions and representational constraints.

**Figure 3:**
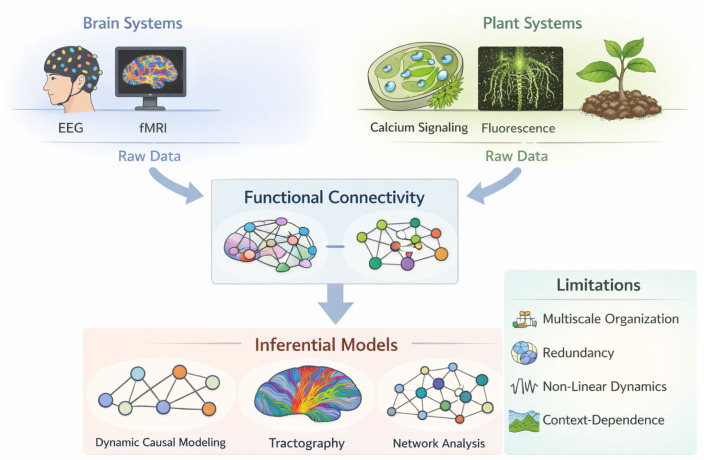
Inferential reconstruction and limitations in distributed biological systems. Biological function is not directly observable but inferred from indirect measurements. Raw data from brain systems (e.g., EEG, fMRI) and plant systems (e.g., calcium signaling, fluorescence imaging) are transformed into representations of functional connectivity. Model-based approaches for functional inference (e.g., dynamic causal modeling, network analysis) are complemented by structural reconstruction methods such as tractography, which provide constraints but do not directly capture functional dynamics. These inferences are constrained by the complexity of biological systems, including multiscale organization, redundancy, non-linear dynamics, and context-dependent interactions, leading to inherent interpretative limitations.

Similarly, in plant biology, signaling networks are reconstructed using gene expression data, imaging of calcium and electrical activity, and perturbation experiments [26]. Cross-talk is inferred from patterns of dependence rather than directly observed. The inferential nature of these approaches and their associated limitations are summarized in Figure 3.

In both fields, scientific explanation involves constructing and testing hypotheses about relational architecture, rather than identifying isolated components. This implies that biological explanation is inherently model-dependent, insofar as the inferred organization of a system partly reflects the assumptions, resolution limits, and representational constraints of the methods used to reconstruct it. Consequently, different analytical frameworks may yield partially different functional architectures for the same underlying biological process [9,43]. This highlights that network models provide indirect reconstructions of functional organization, rather than direct observations of underlying biological processes.

Recent methodological developments further support the importance of temporally structured coordination dynamics in biological systems. In neuroscience, machine learning and AI-driven approaches are increasingly being used to reconstruct dynamic functional architectures from large-scale neuroimaging data, allowing the identification of transient brain states and adaptive coordination regimes [7,8,10,44,45]. Similarly, recent advances in plant electrophysiology and live-cell imaging enable real-time tracking of systemic signaling dynamics across tissues [28,29,30]. These developments provide increasingly precise tools for distinguishing interactions that are merely present from those that become functionally consequential under specific physiological contexts [9,44].

A direct methodological implication of this framework is that experimental designs focusing exclusively on regional activation or static connectivity may systematically miss the functional organization of biological systems. Instead, approaches should prioritize the identification of temporally structured interaction patterns and their causal relevance for system-level outcomes. This shift entails integrating time-resolved analyses with perturbational strategies, enabling the distinction between interactions that are merely present and those that are functionally effective. In this sense, the study of biological function requires not only mapping components or connections, but reconstructing the conditions under which specific interaction patterns are selectively stabilized and become behaviorally consequential.

## 7. Limitations and Future Directions

A central challenge for the coordination framework concerns its empirical operationalization. To move beyond a conceptual proposal, coordination must be distinguished from related constructs such as connectivity strength, network topology, or undifferentiated temporal variability. In this perspective, coordination can be quantified as the selective stabilization of interaction patterns that predict functional outcomes across contexts. This requires integrating time-resolved analyses (e.g., state-based or sliding-window approaches) with perturbational strategies that assess the causal relevance of specific interactions [5,6].

The coordination framework generates a set of empirically testable and potentially falsifiable predictions. First, functional outcomes should be better predicted by the configuration and temporal sequencing of interactions than by the activity of individual components or by static measures of connectivity alone [5]. Second, perturbations targeting integrative or hub-like elements should disproportionately affect system-level function, even when average connectivity strength or local activity levels remain relatively preserved [17]. Third, similar functional outcomes may arise from different configurations of interacting components, provided that they instantiate equivalent coordination regimes, reflecting context-dependent degeneracy at the level of interaction patterns [13]. Fourth, biological systems should exhibit signatures of metastability, characterized by the emergence, stabilization, and dissolution of transient coordination regimes over time, rather than convergence to fixed functional states [5,6]. In this context, metastability refers to the coexistence of tendencies toward both integration and segregation, allowing biological systems to flexibly transition between partially stable coordination states without converging to a fixed global configuration [6,11].

Importantly, the coordination account would be challenged if time-resolved interaction patterns fail to provide additional explanatory or predictive power beyond established measures of static or dynamic connectivity [4]. Conversely, the framework predicts that models incorporating the selective configuration and temporal structuring of interactions should outperform those based solely on regional activity or aggregate connectivity strength in predicting behavior across contexts.

Despite its explanatory potential, the coordination-based framework presents several limitations. Emphasizing interactions risks underestimating the role of component-level constraints, potentially reducing mechanistic specificity. In addition, current methodological approaches remain limited in their ability to distinguish causal from correlational interactions, particularly in complex, multiscale systems [9,19]. Finally, the reliance on model-based inference raises the broader question of how accurately reconstructed interaction patterns reflect underlying biological processes.

Future research should aim to integrate multi-scale data and improve causal inference methods, bridging structural constraints with dynamic coordination. More generally, experimental approaches should prioritize the identification of temporally structured interaction patterns and their causal contribution to system-level function, rather than focusing exclusively on isolated components or static connectivity maps. Within this framework, biological responses to perturbation are best understood as system-wide reorganizations of interaction patterns, rather than as linear propagation along predefined pathways.

## 8. Conclusions

Contemporary neuroscience and systems biology have already moved substantially beyond strictly localizationist accounts of biological function by emphasizing distributed and network-based explanations of behavior and adaptation. However, distributed organization alone does not fully explain how coherent biological functions emerge across changing contexts. The central argument of this review is that biological function is best understood as arising from the selective stabilization of interactions among distributed components, forming transient coordination regimes that become causally relevant for specific outcomes.

The comparison between brain networks and plant signaling systems illustrates how complex adaptive behavior can arise without centralized control despite substantial differences in biological substrate, signaling mechanisms, and evolutionary history. Although these systems are not mechanistically equivalent, both demonstrate that coherent functional outcomes depend on the coordination of distributed processes rather than on the isolated activity of individual components.

The proposed framework also generates empirically testable predictions. If coordination constitutes a distinct explanatory principle, then temporally structured interaction patterns should provide greater explanatory and predictive power than localized activity or static measures of connectivity alone. Future research should therefore prioritize time-resolved analyses, perturbational approaches, and multiscale methods capable of identifying which interactions become selectively stabilized and causally relevant under specific conditions.

The novelty of the present proposal does not lie in identifying new forms of biological dynamics, but in treating coordination itself as the primary explanatory unit of biological function. From this perspective, the fundamental question of biological explanation becomes not where function is located, but how function is coordinated.

## Figures and Tables

**Table 1:** Comparative overview of major explanatory frameworks of biological organization and the specific contribution of the proposed coordination framework.

Framework	Primary Explanatory Focus	Strengths	Limitations	Contribution of Coordination Framework
Localizationism	Specialized anatomical regions and structures	Mechanistic specificity	Limited account of flexibility and degeneracy	Emphasizes interaction-dependent function
Network neuroscience	Connectivity architecture and large-scale networks	Distributed organization	Often descriptive of connectivity patterns	Focus on selective stabilization of interactions
Systems biology	Emergent behavior from interacting components	Multiscale integration	Limited temporal specificity	Prioritizes temporally structured coordination regimes
Coordination framework	Context-dependent selective organization of interactions	Explains adaptive functional emergence	Requires advanced time-resolved causal inference	Integrates causal relevance, temporal structure, and metastability

**Table 2:** Representative empirical evidence supporting coordination-based accounts of biological function in brain and plant systems.

Domain	Study	Main Finding	Relevance to Coordination Framework
Brain	Vidaurre et al. (2017) [10]	Brain activity is organized into recurrent network states evolving across multiple temporal scales.	Supports the temporal organization of coordination regimes.
Brain	Shine et al. (2019) [8]	Cognitive performance is associated with dynamic transitions between integrated and segregated network states.	Function emerges from transient coordination among distributed regions.
Brain	Novelli and Razi (2022) [38]	Edge-centric analyses reveal continuously reconfiguring interaction patterns.	Highlights dynamic interaction structures beyond static connectivity.
Brain	Liu et al. (2022) [39]	Structure–function relationships vary across time and cognitive conditions.	Demonstrates context-dependent coordination dynamics.
Brain	Luppi et al. (2022) [40]	Higher cognition depends on distributed synergistic information integration.	Supports emergent function through coordinated interactions.
Brain	Seguin et al. (2023) [36]	Brain communication depends on dynamic network interactions.	Emphasizes communication and coordination as explanatory principles.
Brain	Fotiadis et al. (2024) [37]	Structure–function coupling reflects distributed network organization.	Supports relational rather than purely localized explanations.
Plant	Toyota et al. (2018) [24]	Long-distance calcium waves mediate systemic defense signaling.	Demonstrates distributed organism-wide coordination.
Plant	Farmer et al. (2020) [28]	Electrical signaling regulates systemic hormonal responses.	Illustrates integration across signaling modalities.
Plant	Fichman et al. (2019) [42]	ROS signaling forms part of a whole-plant communication network.	Supports coordinated multi-signal integration across tissues.

## Data Availability

No new data were created or analyzed in this study. Data sharing is not applicable to this article.

## Abbreviations

The following abbreviations are used in this manuscript:

AI	Artificial Intelligence
DCM	Dynamic Causal Modeling
DSI	Diffusion Spectrum Imaging
DTI	Diffusion Tensor Imaging
EEG	Electroencephalography
fMRI	Functional Magnetic Resonance Imaging
MEG	Magnetoencephalography
MRI	Magnetic Resonance Imaging
PLV	Phase-Locking Value
ROS	Reactive Oxygen Species
